# Trends in Diet Quality Among Older US Adults From 2001 to 2018

**DOI:** 10.1001/jamanetworkopen.2022.1880

**Published:** 2022-03-11

**Authors:** Tingxi Long, Kehan Zhang, Yan Chen, Chenkai Wu

**Affiliations:** 1 Global Health Research Center, Duke Kunshan University, Kunshan, Jiangsu, China

## Abstract

**Importance:**

Poor diet is a major risk factor for chronic diseases, disability, and death in the US. As older adults comprise the fastest-growing population segment in the US, it is crucial to describe dietary trends among older adults to identify opportunities and challenges for improving health in old age.

**Objective:**

To characterize trends in overall dietary quality and key food components and nutrients among older US adults by age, sex, race and ethnicity, marital status, educational level, and income.

**Design, Setting, and Participants:**

This serial cross-sectional study used 24-hour dietary recall data from 10 837 adults aged 65 years or older in 9 National Health and Nutrition Examination Survey cycles (2001-2002 to 2017-2018). Statistical analysis was conducted from June 1 to October 1, 2021.

**Exposures:**

Calendar year and sociodemographic subgroups.

**Main Outcomes and Measures:**

Survey-weighted, energy-adjusted mean diet scores and proportions of older US adults with poor, intermediate, or ideal diet scores based on the American Heart Association (AHA) 2020 Strategic Impact Goals for diet. Additional outcomes included the AHA secondary score and the Healthy Eating Index (HEI)–2015 score.

**Results:**

A total of 10 837 individuals (5423 women [50.0%]; 6339 White participants [58.5%]; mean [SD] age, 73.9 [0.1] years) were analyzed. Overall dietary quality deteriorated from 2001 to 2018 among older adults. The mean primary AHA score (total = 50 points) decreased from 19.84 (95% CI, 19.40-20.29) to 18.28 (95% CI, 17.84-18.73; a decrease of 7.9%; *P* < .001 for trend). The mean secondary AHA score (total = 80 points) decreased from 34.75 (95% CI, 34.11-35.39) to 31.83 (95% CI, 31.17-32.48; a decrease of 8.4%; *P* < .001 for trend). The mean HEI-2015 score (total = 100 points) decreased from 47.82 (95% CI, 47.11-48.52) to 45.25 (95% CI, 44.53-45.98; a decrease of 5.4%; *P* < .001 for trend). Based on the primary AHA score, the proportion of US older adults with a poor diet quality significantly increased from 50.9% to 60.9%, the proportion with an intermediate diet quality significantly decreased from 48.6% to 38.7%, and the proportion with ideal diet quality remained consistently low (0.4% in both 2001-2002 and 2017-2018). Dietary variations were identified by sociodemographic subgroups. We found a significant decreasing trend in diet scores among both sexes and all age groups except for those aged 75 to 79 years.

**Conclusions and Relevance:**

The findings of this cross-sectional study suggest that dietary quality worsened for most dietary components among older US adults between 2001 and 2018. Despite improvement in some dietary components, more than half of older US adults still have poor dietary quality.

## Key Points

**Question:**

What are the national trends in overall diet quality and in individual dietary components among older US adults between 2001 and 2018?

**Findings:**

In this cross-sectional study of 10 837 adults aged 65 years or older in the National Health and Nutrition Examination Survey, the mean primary American Heart Association score had a significant 8% decrease. The proportion of older US adults with poor diet quality significantly increased from 51% to 61%, and the proportion with intermediate diet quality significantly decreased from 49% to 39%; the proportion of older US adults with ideal diet quality remained consistently low.

**Meaning:**

These findings suggest that diet quality decreased among older US adults from 2001 to 2018.

## Teaser

This cross-sectional study uses data from the National Health and Nutrition Examination Survey to characterize trends in overall dietary quality and key food components and nutrients among older US adults, by age, sex, race and ethnicity, marital status, educational level, and income.

## Introduction

Poor diet quality is a major risk factor for chronic diseases, disability, frailty, and death among older adults.^1,2,3^ The 2015-2020 Dietary Guidelines for Americans^4^ have provided recommendations that encourage healthy eating patterns and regular physical activity to maintain good health and reduce the risk of chronic disease for individuals of all ages. Older adults are the fastest-growing segment of the population in the US. The number of adults aged 65 years or older will more than double by 2060, accounting for nearly one-fourth of the total US population.^5^ A healthy diet is crucial for maintaining older adults’ physical and mental health.^6,7,8,9^ Understanding the trends of dietary quality among older adults in the United States could assist with the informed implementation of evidence-based policies and dietary interventions to improve dietary quality and reduce diet-related morbidity and mortality in old age. Investigation of whether and how such trends vary across sociodemographic subgroups could uncover disparities in dietary quality and identify high-risk individuals.

Rehm et al^10^ characterized the trends of both dietary patterns and a wide range of individual dietary components among adults aged 20 years or older in the US from 1999 to 2012; they found a generally improving diet quality with disparities by race and ethnicity, educational level, and income. However, specific investigation is lacking for older US adults, to our knowledge. In addition, differences in trends of dietary quality and individual food components across population subgroups remain unclear.

To address these research gaps, we used demographic and dietary data from 9 consecutive 2-year cycles from 2001-2002 to 2017-2018 of the National Health and Nutrition Examination Survey (NHANES) to examine temporal changes in both overall diet quality and individual foods and nutrients among US adults aged 65 years or older. We also described the trends in dietary quality and in individual foods and nutrients by sociodemographic characteristics.

## Methods

### Data and Participants

The NHANES is an ongoing, repeated cross-sectional study of a nationally representative sample of noninstitutionalized residents in the US. The primary aim of the NHANES is to evaluate the health and nutritional status of US adults and children by collecting information about their sociodemographic characteristics, lifestyles, and health conditions through interviews, physical examinations, and laboratory tests. Participants are selected through a 4-stage probability sampling design: county level (primary sampling units), census block level, household level, and individual level. The NHANES protocol was approved by the Centers for Disease Control and Prevention and the National Center for Health Statistics ethics review board. All study participants provided written informed consent. The Duke Kunshan University institutional review board determined that this study did not require review because the data are deidentified and available publicly. This study followed the Strengthening the Reporting of Observational Studies in Epidemiology (STROBE) reporting guideline. Further details about the recruitment strategies and study design have been documented elsewhere.^11^

The NHANES Dietary Interview Component includes a 24-hour dietary recall interview and postrecall questionnaire aiming to estimate total intake of food energy (calories), nutrients, and nonnutrient food components from foods and beverages that were consumed during the 24-hour period prior to the interview (midnight to midnight). The present study used data across 9 NHANES cycles (2001-2002 to 2017-2018), including 10 837 US adults aged 65 years or older who were eligible and completed the 24-hour dietary recall. The US Department of Agriculture Automated Multiple-Pass dietary interview was used to collect the 24-hour dietary recalls at the mobile examination center. Intakes of individual food components were energy adjusted using the residual method to evaluate trends in diet quality.^2^

### American Heart Association Diet Score

The American Heart Association (AHA) diet score is a composite indicator of diet quality constructed based on the AHA 2020 Strategic Impact Goals for diet.^12^ The primary score ranges from 0 (lowest) to 50 (highest) and consists of the following 5 components: fruits and vegetables, whole grains, fish and shellfish, sugar-sweetened beverages, and sodium (eTable 1 in the Supplement). The secondary score ranges from 0 (lowest) to 80 (highest) and has 3 additional components: nuts, seeds, and legumes; processed meat; and saturated fat. We scored each beneficial dietary component from 0 to 10 (where 0 indicates the minimum standard and 10 indicates the maximum standard) and each harmful dietary component from 10 to 0 (where more consumption of harmful food is related to a lower score). We also classified the continuous AHA diet score into 3 categories according to the AHA 2020 Strategic Impact Goals for diet.^12^ Poor diet was defined as less than 40% adherence to the AHA 2020 Strategic Impact Goals (<20 for the primary AHA diet score and <32 for the secondary AHA diet score), intermediate diet was defined as 40% to 79.9% adherence (20-39.9 for the primary AHA diet score and 32-63.9 for the secondary AHA diet score), and ideal diet was defined as 80% or higher adherence (40-50 for the primary AHA diet score and 64-80 for the secondary AHA diet score).

### Healthy Eating Index

The Healthy Eating Index (HEI)^13^ is a commonly used composite measure of diet quality and assesses how well dietary components align with the US Department of Agriculture 2015-2020 Dietary Guidelines for Americans.^4^ We used the HEI-2015, which is the latest version of the HEI that corresponds to the 2015-2020 Dietary Guidelines for Americans. The HEI-2015 score ranges from 0 (lowest) to 100 (highest). A total of 13 dietary components are included, with 9 beneficial components (total fruits, whole fruits, total vegetables, greens and beans, whole grains, dairy, total protein foods, seafood and plant proteins, and fatty acids) and 4 harmful components (refined grains, sodium, added sugar, and saturated fats). We calculated the HEI-2015 score using the population ratio method recommended by the National Cancer Institute and the US Department of Agriculture (eTable 2 in the Supplement).^6^

### Sociodemographic Characteristics

Sociodemographic characteristics were collected using computer-assisted interviews by trained NHANES interviewers in the home. Self-reported characteristics included sex, age (65-69, 70-74, 75-79, or ≥80 years), race and ethnicity (Mexican American, non-Hispanic Black, non-Hispanic White, other Hispanic, or other [including non-Hispanic Asian and multiracial individuals]), educational level (below high school, high school or General Educational Development certification, some college, or college graduate or above), marital status (married or living together, widowed, divorced or separated, or never married), and ratio of family income to poverty level calculated by dividing family or individual income by the Department of Health and Human Services poverty guidelines (<1.30, 1.30-1.84, 1.85-3.0, or >3.0). We merged the other Hispanic group into the Mexican American group owing to small sample size.

### Statistical Analysis

Statistical analysis was conducted from June 1 to October 1, 2021. We accounted for the complex, multistage probability sampling design of the NHANES by specifying the sampling weight, strata, and primary sampling unit parameters to obtain nationally representative estimates. We estimated the mean for the primary AHA diet score (range, 0-50), the secondary AHA diet score (range, 0-80), the HEI-2015 score (range, 0-100), and each component diet score for each NHANES cycle. We estimated the proportions of 3 categories of diet quality (poor, intermediate, and ideal). We considered the survey year as a continuous variable in a survey-weighted linear regression model to examine the statistical significance of trends in diet quality over time. We calculated both absolute and relative differences in estimated mean values of diet scores between 2001-2002 and 2017-2018 NHANES cycles in survey-weighted linear regression models, in which the survey year was treated as a categorical variable (reference group, 2001-2002 cycle). To evaluate the statistical significance of differences in diet scores over time, we used a survey-weighted Wald *F* test to evaluate a multiplicative interaction term between survey year as a continuous variable and each sociodemographic characteristic as a categorical variable. Participants with missing data on educational level (n = 26 [0.2%]) or marital status (n = 4 [0.04%]) were excluded from subgroup analysis.

Tests were 2-sided with a significance level of *P* < .05. Stata, version 15.6 (StataCorp LLC) and R Studio, version 4.0.1 (R Group for Statistical Computing) were used for statistical analyses. No adjustments were made for multiple comparisons; the findings of secondary analyses should be interpreted as exploratory.

## Results

### Participant Characteristics

A total of 10 837 adults (5423 women [50.0%]; 6339 White participants [58.5%]; mean [SD] age, 73.9 [0.1] years) aged 65 years or older who completed the first valid 24-hour dietary recall were included (85.6% completed the first dietary recall). From 2001-2002 to 2017-2018, the proportion of adults aged 65 to 69 years increased from 30.8% to 35.4%, while adults aged 80 years or older decreased from 23.3% to 19.3% (Table 1). Non-Hispanic White individuals decreased from 84.2% in 2001-2002 to 77.7% in 2017-2018, while individuals of other races and ethnicities increased from 1.3% in 2001-2002 to 5.8% in 2017-2018. Widowed participants decreased from 32.9% to 22.2% over the 18 years. Socioeconomic status improved over time. From 2001-2002 to 2017-2018, older adults with a college degree or higher increased from 20.0% to 34.5%; the proportion of persons with a ratio of family income to poverty level of less than 1.30 decreased from 24.7% to 11.5%.

**Table 1.** Sociodemographic Characteristics of US Adults Aged 65 Years or Older by National Health and Nutrition Examination Survey Cycles, 2001-2018

Characteristic^a^	Count, No. (survey-weighted %)^b^								
	2001-2002	2003-2004	2005-2006	2007-2008	2009-2010	2011-2012	2013-2014	2015-2016	2017-2018
No.	1155	1296	1047	1396	1379	1030	1105	1209	1220
Age group, y									
65-69	292 (30.8)	328 (30.3)	285 (31.8)	373 (30.2)	382 (32.8)	307 (32.4)	367 (36.2)	378 (37.5)	388 (35.4)
70-74	292 (25.3)	310 (26.8)	263 (26.3)	364 (24.5)	370 (25.8)	258 (27.2)	293 (26.6)	302 (24.4)	317 (27.3)
75-79	208 (20.6)	243 (20.2)	188 (19.5)	301 (21.0)	255 (17.3)	186 (16.6)	174 (16.0)	219 (17.1)	207 (18.1)
≥80	363 (23.3)	415 (22.7)	311 (22.4)	358 (24.3)	372 (24.1)	279 (23.7)	271 (21.2)	310 (21.0)	308 (19.3)
Sex									
Female	585 (56.6)	650 (55.8)	499 (56.8)	708 (56.3)	691 (54.8)	506 (54.1)	575 (54.2)	615 (56.6)	594 (54.7)
Male	570 (43.4)	646 (44.2)	548 (43.2)	688 (43.7)	688 (45.2)	524 (45.9)	530 (45.8)	594 (43.4)	626 (45.3)
Race and ethnicity									
Hispanic	200 (7.0)	270 (5.5)	133 (4.5)	258 (6.4)	260 (6.5)	148 (6.2)	187 (6.7)	334 (7.5)	196 (7.9)
Non-Hispanic									
Black	171 (7.5)	163 (7.9)	200 (8.5)	241 (8.0)	213 (8.1)	256 (8.4)	198 (7.6)	196 (7.2)	272 (8.5)
White	770 (84.2)	830 (83.2)	692 (84.4)	862 (82.6)	856 (81.6)	527 (79.6)	624 (80.7)	565 (78.6)	613 (77.7)
Other^c^	14 (1.3)	33 (3.3)	22 (2.6)	35 (3.1)	50 (3.7)	99 (5.8)	96 (5.0)	114 (6.7)	139 (5.8)
Educational level									
Total No.	1152	1290	1046	1393	1375	1027	1104	1207	1215
<High school diploma	449 (30.4)	533 (29.0)	380 (27.2)	539 (28.5)	477 (24.7)	256 (23.1)	279 (16.9)	353 (15.2)	280 (12.3)
High school graduate or GED	269 (27.2)	321 (30.8)	293 (30.7)	364 (29.3)	307 (24.5)	226 (22.2)	259 (21.9)	265 (22.6)	299 (27.2)
Some college	249 (22.4)	251 (22.4)	209 (22.4)	260 (21.5)	336 (27.4)	256 (29.7)	298 (31.4)	334 (32.7)	361 (26.2)
College degree or higher	185 (20.0)	185 (17.8)	164 (19.7)	230 (20.7)	255 (23.4)	189 (25.0)	268 (29.8)	255 (29.6)	275 (34.5)
Marital status									
Married or living with partner	751 (54.6)	804 (57.2)	631 (56.2)	831 (56.8)	853 (61.9)	667 (60.7)	716 (64.0)	737 (59.6)	822 (60.4)
Widowed	523 (32.9)	503 (30.9)	381 (30.1)	456 (28.9)	452 (26.7)	357 (24.3)	349 (24.1)	333 (22.1)	350 (22.2)
Divorced or separated	135 (9.2)	132 (8.8)	137 (10.9)	196 (11.0)	166 (8.5)	162 (11.2)	187 (12.0)	239 (14.1)	262 (14.8)
Never married	47 (3.3)	54 (3.1)	36 (2.4)	72 (3.3)	52 (2.9)	61 (3.7)	53 (3.4)	68 (4.0)	63 (2.5)
Ratio of family income to poverty level^d^									
Total No.	1061	1218	978	1260	1252	928	1035	1042	1072
<1.30^e^	306 (24.7)	357 (20.0)	254 (18.8)	361 (19.5)	341 (18.5)	317 (20.4)	306 (19.0)	338 (18.4)	237 (11.5)
1.30 to <1.85	165 (13.6)	212 (15.6)	165 (15.2)	234 (18.1)	183 (12.5)	140 (12.6)	144 (13.0)	184 (12.4)	222 (12.8)
1.85 to <3.00	251 (23.9)	289 (26.6)	244 (27.2)	317 (27.8)	272 (22.2)	181 (23.8)	203 (20.6)	202 (18.8)	242 (21.3)
≥3.00^f^	339 (37.8)	360 (37.8)	315 (38.8)	348 (34.6)	456 (46.9)	290 (43.2)	382 (47.4)	318 (50.3)	371 (54.3)

Abbreviation: GED, General Educational Development certification.

^a^ The numbers may not sum to the total number of participants owing to missing data.

^b^ The percentages were weighted.

^c^ Includes non-Hispanic Asian and multiracial individuals.

^d^ Represents the ratio of family income to the federal poverty threshold, adjusting for household size. For reference, the federal threshold in 2012 for a family of 4 was $23 492 per year. A family of 4 earning $42 460 per year would have a ratio of 1.85.

^e^ A lower level of income.

^f^ A higher level of income.

### Trends in Diet Quality

**Figure 1.**
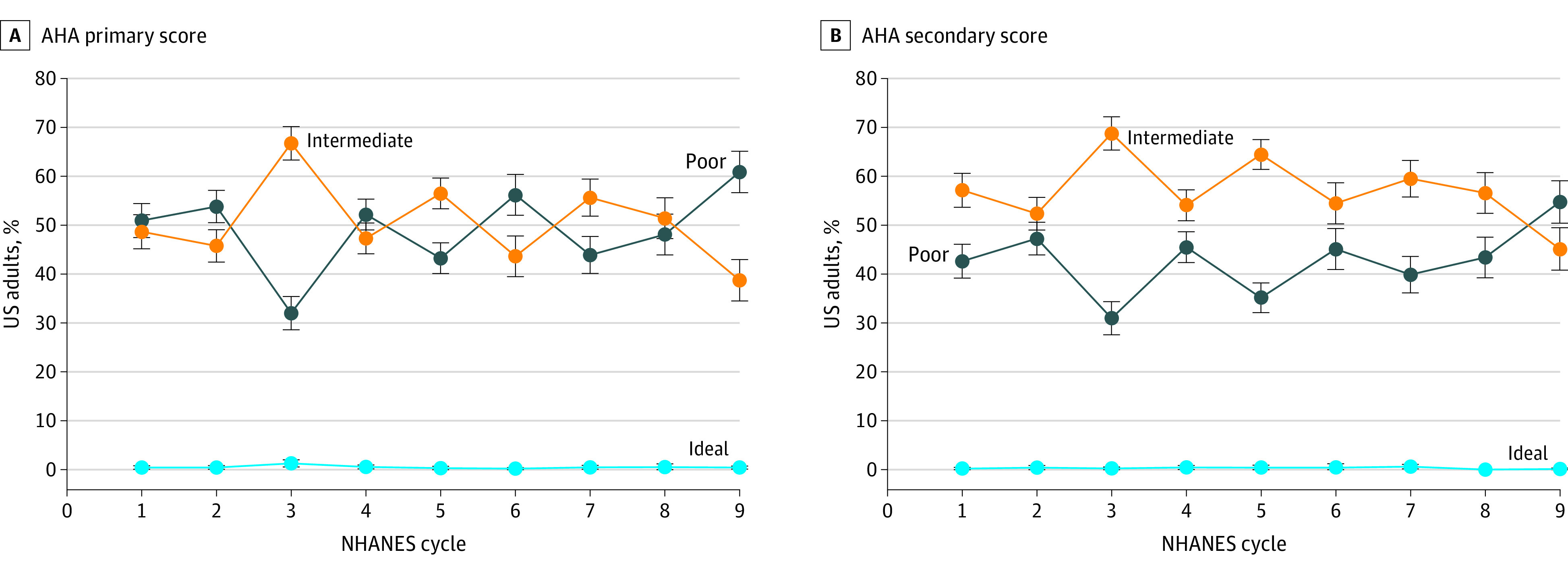
Trends in Estimated Proportions of US Adults Aged 65 Years or Older With Poor, Intermediate, or Ideal Diet Quality, National Health and Nutrition Examination Survey (NHANES) Cycles, 2001-2018 AHA indicates American Heart Association. Error bars indiate 95% CIs.

From 2001-2002 to 2017-2018, overall diet quality decreased based on the primary AHA diet score, secondary AHA diet score, and the HEI-2015 score (Table 2). The mean primary AHA diet score decreased from 19.84 (95% CI, 19.40-20.29) to 18.28 (95% CI, 17.84-18.73) of 50 (a decrease of 7.9%; *P* < .001 for trend), the mean secondary AHA diet score decreased from 34.75 (95% CI, 34.11-35.39) to 31.83 (95% CI, 31.17-32.48) of 80 (a decrease of 8.4%; *P* < .001 for trend), and the mean HEI-2015 total score decreased from 47.82 (95% CI, 47.11-48.52) to 45.25 (95% CI, 44.53-45.98) of 100 (a decrease of 5.4%; *P* < .001 for trend; eFigure in the Supplement). Based on the primary AHA diet score, the proportion of older US adults with poor diet quality significantly increased from 50.9% to 60.9%, the proportion with intermediate diet quality significantly decreased from 48.6% to 38.7%, and the proportion with ideal diet quality remained consistently low across all years (0.4% in both 2001-2002 and 2017-2018) (Figure 1).

**Table 2.** Mean Diet Quality Scores Among US Adults Aged 65 Years or Older by National Health and Nutrition Examination Survey Cycles, 2001-2018

AHA and HEI-2015 total scores	Survey-weighted mean score (95% CI)									*P* value for trend
	2001-2002	2003-2004	2005-2006	2007-2008	2009-2010	2011-2012	2013-2014	2015-2016	2017-2018	
No.	1155	1296	1047	1396	1379	1030	1105	1209	1220	
AHA scores										
Primary score	19.84 (19.40-20.29)	19.19 (18.77-19.62)	24.00 (23.53-24.48)	19.62 (19.20-20.04)	21.04 (20.64-21.45)	18.66 (18.19-19.14)	21.55 (21.09-22.01)	20.36 (19.91-20.80)	18.28 (17.84-18.73)	<.001
Fruits and vegetables	5.07 (4.87-5.28)	4.39 (4.19-4.58)	3.74 (3.52-3.95)	4.55 (4.37-4.74)	4.54 (4.36-4.73)	3.86 (3.65-4.08)	3.22 (3.01-3.43)	3.71 (3.51-3.91)	3.71 (3.52-3.91)	<.001
Whole grains	1.76 (1.59-1.93)	1.49 (1.34-1.64)	1.56 (1.40-1.73)	1.89 (1.73-2.04)	1.51 (1.36-1.65)	1.64 (1.46-1.82)	1.63 (1.46-1.80)	1.34 (1.18-1.49)	1.51 (1.34-1.67)	.02
Fish and shellfish	2.61 (2.45-2.78)	1.12 (0.96-1.29)	6.84 (6.64-7.04)	0.95 (0.80-1.10)	3.98 (3.81-4.15)	1.23 (1.04-1.43)	3.97 (3.79-4.16)	2.92 (2.75-3.09)	1.77 (1.59-1.95)	<.001
Sugar-sweetened beverages	5.08 (4.84-5.33)	6.63 (6.40-6.85)	5.82 (5.56-6.09)	6.56 (6.34-6.78)	5.93 (5.70-6.16)	6.02 (5.76-6.29)	6.53 (6.28-6.78)	6.93 (6.70-7.17)	5.74 (5.49-5.98)	.03
Sodium	5.31 (5.16-5.46)	5.56 (5.42-5.70)	6.04 (5.88-6.21)	5.67 (5.53-5.81)	5.08 (4.95-5.21)	5.91 (5.75-6.06)	6.20 (6.05-6.36)	5.45 (5.31-5.60)	5.55 (5.41-5.70)	.03
Secondary score	34.75 (34.11-35.39)	33.84 (33.21-34.47)	38.12 (37.43-38.81)	33.94 (33.34-34.55)	36.16 (35.57-36.74)	33.67 (33.02-34.33)	35.56 (34.88-36.23)	35.10 (34.44-35.75)	31.83 (31.17-32.48)	<.001
Nuts, seeds, and legumes	3.07 (2.91-3.22)	1.69 (1.51-1.87)	1.85 (1.68-2.02)	2.39 (2.22-2.55)	3.39 (3.24-3.54)	1.71 (1.56-1.86)	1.29 (1.13-1.45)	2.24 (2.07-2.41)	1.81 (1.65-1.98)	<.001
Processed meat	6.02 (5.78-6.26)	7.50 (7.28-7.71)	7.16 (6.91-7.42)	6.56 (6.34-6.77)	6.32 (6.10-6.55)	7.52 (7.27-7.76)	7.43 (7.19-7.67)	7.49 (7.26-7.72)	7.07 (6.82-7.31)	.002
Saturated fat	5.82 (5.62-6.02)	5.47 (5.28-5.66)	5.11 (4.89-5.32)	5.38 (5.20-5.57)	5.40 (5.21-5.59)	5.78 (5.57-5.99)	5.28 (5.07-5.50)	5.01 (4.80-5.21)	4.66 (4.46-4.87)	<.001
HEI-2015 total score	47.82 (47.11-48.52)	47.03 (46.35-47.71)	46.37 (45.61-47.13)	46.94 (46.28-47.60)	46.17 (45.51-46.83)	46.26 (45.50-47.03)	46.26 (45.47-47.05)	43.73 (42.95-44.52)	45.25 (44.53-45.98)	<.001
Adequacy components										
Total fruits	3.10 (2.98-3.23)	2.97 (2.85-3.09)	3.06 (2.93-3.19)	2.95 (2.83-3.07)	3.02 (2.91-3.14)	2.85 (2.71-2.99)	2.77 (2.64-2.90)	2.65 (2.53-2.78)	2.44 (2.32-2.57)	<.001
Whole fruits	3.34 (3.21-3.47)	3.24 (3.11-3.36)	3.30 (3.16-3.44)	3.09 (2.96-3.21)	3.21 (3.08-3.33)	3.00 (2.85-3.14)	2.93 (2.79-3.07)	2.86 (2.72-2.99)	2.62 (2.49-2.76)	<.001
Total vegetables	3.17 (3.06-3.29)	3.09 (2.98-3.19)	3.05 (2.93-3.16)	3.09 (2.98-3.19)	3.00 (2.89-3.10)	2.87 (2.75-2.99)	2.90 (2.79-3.02)	2.71 (2.60-2.83)	2.68 (2.57-2.80)	<.001
Greens and beans	1.41 (1.28-1.53)	1.44 (1.32-1.56)	1.37 (1.24-1.50)	1.40 (1.28-1.51)	1.44 (1.32-1.55)	1.28 (1.15-1.41)	1.49 (1.37-1.62)	1.47 (1.35-1.59)	1.34 (1.22-1.46)	.15
Whole grains	2.24 (2.03-2.44)	2.05 (1.86-2.23)	2.06 (1.85-2.26)	2.17 (1.99-2.36)	2.17 (1.99-2.36)	2.25 (2.03-2.48)	2.31 (2.09-2.52)	1.77 (1.58-1.97)	1.78 (1.59-1.96)	.005
Dairy	5.65 (5.41-5.88)	5.32 (5.10-5.54)	5.72 (5.47-5.96)	5.60 (5.39-5.81)	5.34 (5.12-5.56)	5.26 (5.00-5.51)	4.87 (4.64-5.11)	4.71 (4.48-4.95)	4.34 (4.12-4.57)	<.001
Total protein foods	2.86 (2.74-2.98)	2.86 (2.74-2.97)	2.80 (2.68-2.92)	2.69 (2.58-2.80)	2.78 (2.67-2.89)	3.09 (2.96-3.21)	3.15 (3.04-3.27)	3.09 (2.97-3.21)	3.08 (2.97-3.20)	<.001
Seafood and plant proteins	1.31 (1.19-1.44)	1.46 (1.34-1.58)	1.45 (1.31-1.58)	1.41 (1.30-1.53)	1.51 (1.39-1.62)	1.48 (1.35-1.61)	1.47 (1.34-1.60)	1.83 (1.70-1.97)	1.49 (1.36-1.61)	.03
Total monounsaturated fatty acids	6.98 (6.81-7.16)	7.02 (6.86-7.18)	6.70 (6.50-6.89)	6.86 (6.70-7.02)	6.78 (6.61-6.95)	7.12 (6.94-7.31)	6.94 (6.76-7.12)	6.84 (6.66-7.02)	6.96 (6.78-7.13)	.03
Moderation components										
Refined grains	3.56 (3.31-3.80)	3.42 (3.19-3.65)	3.19 (2.95-3.43)	3.67 (3.45-3.89)	3.55 (3.33-3.76)	3.24 (2.99-3.49)	3.66 (3.40-3.91)	3.57 (3.33-3.81)	2.63 (2.41-2.85)	<.001
Sodium	4.81 (4.60-5.02)	4.96 (4.76-5.16)	4.87 (4.64-5.10)	4.70 (4.50-4.89)	4.00 (3.82-4.19)	4.14 (3.92-4.35)	4.23 (4.02-4.44)	4.41 (4.21-4.61)	4.71 (4.51-4.91)	.03
Added sugar	2.72 (2.54-2.89)	2.89 (2.72-3.05)	2.84 (2.65-3.03)	3.08 (2.92-3.25)	3.15 (2.98-3.32)	3.09 (2.90-3.28)	3.47 (3.28-3.67)	3.63 (3.45-3.81)	5.69 (5.50-5.88)	<.001
Saturated fats	6.66 (6.47-6.85)	6.34 (6.15-6.52)	5.98 (5.76-6.19)	6.23 (6.05-6.41)	6.22 (6.04-6.41)	6.61 (6.40-6.81)	6.06 (5.85-6.27)	5.81 (5.61-6.01)	5.49 (5.28-5.69)	<.001

Abbreviations: AHA, American Heart Association; HEI, Healthy Eating Index.

### Trends in Individual Components of Diet Scores

**Figure 2.**
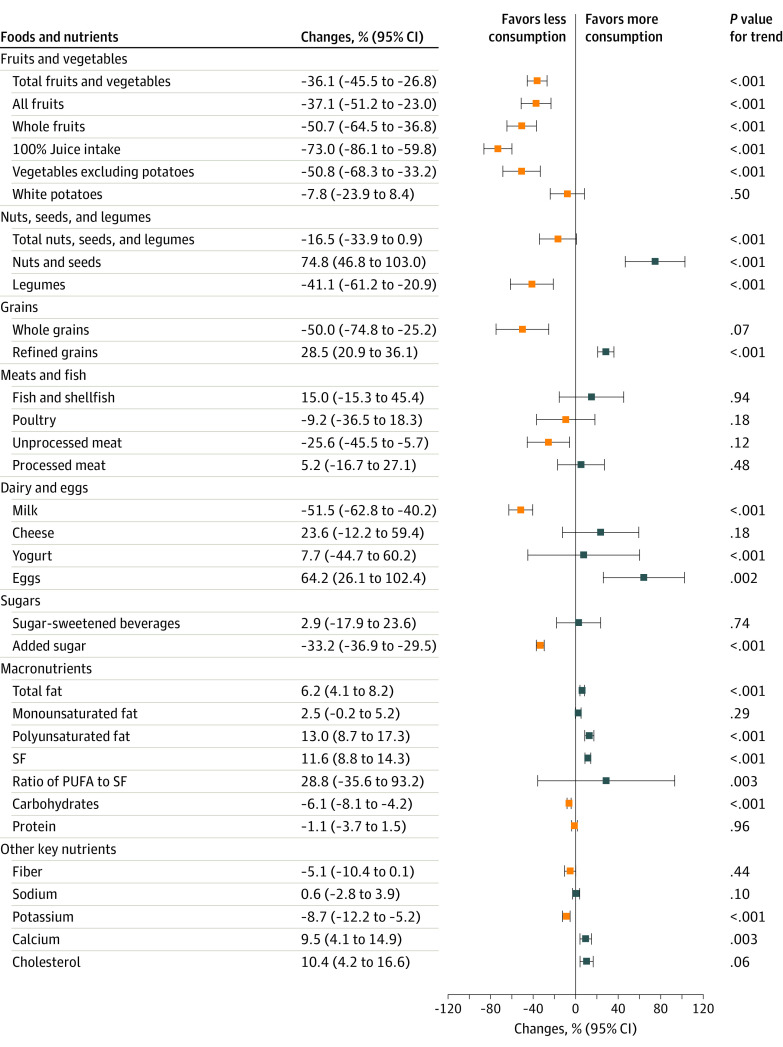
Changes in Estimated Mean Consumption of Dietary Components Among US Adults Aged 65 Years or Older Between 2001-2002 and 2017-2018 PUFA indicates polyunsaturated fatty acid; SF, saturated fat. Error bars indiate 95% CIs.

We found statistically significant changes among individual components of the AHA diet score (Table 2 and Figure 2). From 2001-2002 to 2017-2018, the mean (SD) consumption of total fruits and vegetables significantly decreased from 3.90 (95% CI, 3.62-4.19) to 2.49 (95% CI, 2.13-2.86) servings per day (difference, −36.1% [95% CI, −45.5% to −26.8%]). The mean (SD) consumption of fish and shellfish increased from 0.23 (95% CI, 0.18-0.28) to 0.27 (95% CI, 0.20-0.34) servings per day (difference, 15.0% [95% CI, −15.3% to 45.4%]). The mean (SD) consumption of nuts, seeds, and legumes decreased from 0.37 (95% CI, 0.29-0.46) to 0.31 (95% CI, 0.25-0.38) servings per day (difference, −16.5% [95% CI, −33.9% to 0.9%]. The mean (SD) consumption of processed meat increased from 0.40 (95% CI, 0.35-0.45) to 0.42 (95% CI, 0.33-0.51) servings per day (difference, 5.2% [95% CI, −16.7% to 27.1%]). The mean (SD) consumption of sugar-sweetened beverages increased from 4.37 (95% CI, 3.94-4.81) to 4.50 (95% CI, 3.59-5.41) servings per day (difference, 2.9% [95% CI, −17.9% to 23.6%]). The mean (SD) consumption of saturated fat increased from 0.36 (95% CI, 0.35-0.37) to 0.40 (95% CI, 0.39-0.41) servings per day (difference, 11.6% [95% CI, 8.8%-14.3%]).

Among individual components of the HEI-2015 score, only greens and beans showed no significant change in score between 2001-2002 and 2017-2018 (Table 2). The mean component scores of total protein foods, seafood and plant proteins, and added sugar increased significantly between 2001-2002 and 2017-2018.

Among subcomponents of food groups, the largest change in consumption was among nuts and seeds, which increased 74.8% (95% CI, 46.8%-103.0%) from 2001-2002 to 2017-2018 (Figure 2). However, mean consumption of total nuts, seeds, and legumes significantly decreased because of the substantial reduction in consumption of legumes (−41.1% [95% CI, −61.2% to −20.9%]). All food components in the fruits and vegetables category showed a statistically significant decreasing trend in mean consumption except for white potatoes. Between 2001-2002 and 2017-2018, the mean consumption of total fruits and vegetables decreased by 36.1%, consumption of all fruits decreased by 37.1%, consumption of whole fruits decreased by 50.7%, intake of 100% juice decreased by 73.0%, and consumption of vegetables excluding potatoes decreased by 50.8%.

Most of the specific macronutrients and key nutrient scores had a significantly increasing trend between 2001-2002 and 2017-2018. We observed a 6.2% (95% CI, 4.1%-8.2%) increase in total fat consumption (Figure 2); consumption of polyunsaturated fat increased 13.0% (95% CI, 8.7%-17.3%), and consumpton of saturated fat increased 11.6% (95% CI, 8.8%-14.3%). Consumption of dietary calcium and cholesterol increased 9.5% (95% CI, 4.1%-14.9%) and 10.4% (95% CI, 4.2%-16.6%), respectively. We did not observe significant changes in consumption of fish and shellfish, poultry, unprocessed meat, processed meat, or fiber between 2001 and 2018.

### Subgroup Analysis

We presented the trends for the primary AHA diet scores by sociodemographic characteristics between 2001-2002 and 2017-2018 in Table 3. We observed a significant decreasing trend in primary AHA diet scores among both sexes and all age groups except for individuals aged 75 to 79 years. The decreasing trend in diet quality was most notable among non-Hispanic White individuals (from 19.88 [95% CI, 19.30-20.47] in 2001-2002 to 17.77 [95% CI, 16.96-18.58] in 2017-2018; *P* < .001 for trend); we did not observe a significant change among non-Hispanic Black or Hispanic individuals. Non-Hispanic White individuals had a more significant decrease than non-Hispanic Black individuals in diet quality between 2001-2002 and 2017-2018. Most sociodemographic subgroups experienced a significant decrease in AHA secondary diet scores between 2001-2002 and 2017-2018 (eTable 3 in the Supplement).

**Table 3.** Trends in Estimated Primary AHA Diet Scores by Sociodemographic Characteristics, National Health and Nutrition Examination Survey Cycles, 2001-2018

Group	Survey-weighted AHA primary score, mean (95% CI)^a^									*P* values	
	2001-2002	2003-2004	2005-2006	2007-2008	2009-2010	2011-2012	2013-2014	2015-2016	2017-2018	For trend^b^	For interaction^c^
Age group, y											
65-69	19.63 (18.57-20.70)	17.69 (16.49-18.89)	22.45 (21.30-23.61)	17.64 (16.65-18.64)	19.92 (18.86-20.97)	17.53 (16.01-19.05)	19.89 (18.88-20.89)	19.41 (18.08-20.73)	17.08 (15.74-18.42)	.03	[Reference]
70-74	19.42 (18.43-20.42)	18.86 (17.92-19.80)	23.83 (22.61-25.04)	19.33 (18.43-20.22)	20.78 (19.91-21.66)	19.08 (17.96-20.19)	21.42 (20.31-22.52)	19.27 (18.09-20.45)	18.10 (16.84-19.35)	.01	.89
75-79	19.63 (18.45-20.80)	19.84 (18.69-20.99)	24.02 (22.81-25.23)	20.12 (19.11-21.13)	20.90 (19.97-21.83)	18.17 (16.85-19.48)	21.29 (19.97-22.61)	20.31 (19.00-21.62)	19.49 (18.33-20.66)	.11	.69
≥80	20.59 (19.71-21.48)	21.48 (20.75-22.20)	26.67 (25.85-27.49)	21.54 (20.69-22.39)	23.55 (22.82-24.28)	19.85 (18.95-20.76)	23.46 (22.55-24.37)	21.83 (20.96-22.71)	18.43 (17.48-19.38)	<.001	.22
Sex											
Female	20.54 (19.84-21.25)	19.79 (19.07-20.50)	25.55 (24.80-26.29)	19.98 (19.33-20.63)	22.45 (21.83-23.06)	19.60 (18.75-20.44)	21.97 (21.27-22.68)	21.29 (20.45-22.12)	18.47 (17.59-19.36)	<.001	.79
Male	18.83 (18.07-19.59)	18.68 (17.89-19.47)	22.10 (21.24-22.97)	18.92 (18.20-19.65)	19.65 (18.91-20.39)	17.45 (16.44-18.45)	20.44 (19.57-21.31)	18.41 (17.43-19.39)	17.54 (16.57-18.51)	.001	[Reference]
Race and ethnicity											
Hispanic	19.28 (17.48-21.07)	18.87 (17.47-20.26)	21.81 (20.27-23.36)	19.43 (18.44-20.42)	20.13 (19.18-21.08)	18.05 (16.70-19.40)	19.57 (18.42-20.73)	20.55 (19.68-21.42)	17.86 (16.59-19.13)	.25	.15
Non-Hispanic											
Black	18.70 (17.43-19.97)	18.74 (17.37-20.11)	23.93 (22.81-25.05)	18.77 (17.62-19.92)	20.47 (19.40-21.54)	17.83 (16.84-18.82)	22.72 (21.41-24.03)	20.66 (19.60-21.72)	18.68 (17.53-19.84)	.80	.01
White	19.88 (19.30-20.47)	19.23 (18.63-19.83)	24.15 (23.48-24.81)	19.43 (18.87-19.99)	21.32 (20.76-21.89)	18.62 (17.84-19.40)	21.11 (20.45-21.77)	19.88 (19.08-20.68)	17.77 (16.96-18.58)	<.001	[Reference]
Educational level											
<High school diploma	19.39 (18.45-20.34)	17.91 (17.09-18.74)	23.77 (22.80-24.75)	18.33 (17.54-19.12)	20.15 (19.29-21.01)	17.09 (15.98-18.20)	20.38 (19.16-21.60)	20.88 (19.94-21.82)	16.99 (15.62-18.36)	.09	[Reference]
High school graduate or GED	19.47 (18.47-20.46)	19.80 (18.83-20.77)	23.95 (22.86-25.05)	19.60 (18.69-20.51)	21.09 (20.17-22.00)	18.48 (17.08-19.89)	20.57 (19.55-21.58)	20.06 (18.80-21.32)	17.14 (15.98-18.30)	<.001	.05
Some college	19.77 (18.74-20.80)	19.09 (17.77-20.41)	24.04 (22.88-25.21)	19.81 (18.71-20.91)	21.70 (20.71-22.69)	18.85 (17.74-19.96)	21.74 (20.73-22.76)	19.43 (18.35-20.52)	18.59 (17.37-19.81)	.01	.46
College degree or higher	20.89 (19.67-22.11)	20.98 (19.76-22.20)	24.71 (23.21-26.21)	20.73 (19.57-21.89)	21.69 (20.60-22.77)	19.86 (18.30-21.42)	21.78 (20.64-22.92)	20.25 (18.74-21.75)	18.72 (17.44-20.00)	<.001	.05
Marital status											
Married or living with partner	19.69 (18.97-20.42)	19.31 (18.58-20.03)	23.83 (23.05-24.61)	19.19 (18.53-19.84)	20.79 (20.15-21.44)	18.63 (17.75-19.51)	21.17 (20.43-21.90)	19.84 (18.96-20.71)	18.01 (17.14-18.89)	<.001	[Reference]
Widowed	20.30 (19.44-21.15)	19.89 (19.04-20.73)	25.03 (23.99-26.07)	20.82 (19.96-21.69)	22.53 (21.79-23.28)	19.21 (18.17-20.26)	22.00 (20.94-23.06)	20.41 (19.20-21.62)	18.75 (17.34-20.16)	.007	.99
Divorced or separated	19.34 (17.62-21.06)	17.62 (15.85-19.39)	23.21 (21.54-24.88)	18.87 (17.47-20.26)	20.26 (18.47-22.05)	16.79 (14.93-18.66)	20.35 (18.93-21.76)	20.00 (18.68-21.32)	17.96 (16.64-19.29)	.17	.51
Never married	18.99 (15.96-22.03)	19.37 (16.27-22.48)	25.38 (22.42-28.35)	18.34 (15.64-21.05)	21.47 (19.36-23.59)	21.30 (18.69-23.91)	22.79 (19.62-25.96)	21.94 (17.62-26.26)	15.12 (13.06-17.18)	.62	.67
Ratio of family income to poverty level											
<1.30	19.50 (18.50-20.49)	18.08 (17.16-19.00)	24.67 (23.47-25.87)	18.69 (17.73-19.65)	20.64 (19.66-21.63)	17.87 (16.72-19.01)	21.28 (20.14-22.42)	19.72 (18.74-20.70)	17.83 (16.68-18.97)	.09	[Reference]
1.30 to <1.85	18.99 (17.68-20.29)	18.58 (17.32-19.85)	23.17 (21.78-24.57)	19.80 (18.58-21.01)	20.93 (19.67-22.19)	16.86 (14.70-19.03)	21.14 (19.76-22.51)	18.83 (17.61-20.05)	18.33 (16.89-19.76)	.06	.68
1.85 to <3.00	19.26 (18.24-20.29)	19.83 (18.59-21.07)	24.51 (23.45-25.58)	19.29 (18.31-20.28)	20.58 (19.61-21.54)	19.16 (17.99-20.32)	20.99 (19.80-22.18)	20.45 (19.09-21.81)	17.15 (15.96-18.33)	<.001	.15
≥3.00	20.47 (19.51-21.44)	20.01 (19.09-20.93)	23.67 (22.59-24.75)	20.00 (19.09-20.92)	21.65 (20.80-22.49)	18.85 (17.67-20.03)	21.40 (20.49-22.32)	19.56 (18.34-20.77)	18.25 (17.10-19.40)	<.001	.06

Abbreviations: AHA, American Heart Association; GED, General Educational Development certification.

^a^ Data were weighted to be nationally representative.

^b^ Estimated by treating survey cycle as continuous variable in survey-weighted logistic regression model.

^c^ Calculated using the regression model for interaction term between survey cycle and sociodemographic subgroups.

## Discussion

This study investigated diet quality and its recent trends among older US adults based on AHA and HEI scores. Overall, there was a decreasing trend in dietary quality among older US adults in the past 20 years. The mean primary AHA diet score and the mean secondary AHA diet score showed a decrease of 7.9% and 8.4%, respectively, from 2001 to 2018; the mean HEI score decreased by 5.4% between 2001 and 2018. We analyzed the trend in individual nutrients and food groups and found that the mean consumption of total fruits and vegetables, nuts, seeds, and legumes decreased from 2001 to 2018, while consumption of fish and shellfish, processed meat, and saturated fat showed an increasing trend.

The most notable finding was the overall trend of deterioration in diet quality among older adults. All 3 composite diet scores—the primary AHA diet score, the secondary AHA diet score, and the HEI-2015 score—showed a modest dcrease between 2001 and 2018. The proportion of older US adults with poor diet quality increased by 10%, and the proportion with an ideal diet quality remained consistently low. Determinants of healthy eating behavior are complex and multifaceted among older adults. Social isolation and loneliness might create difficulties in acquiring and preparing food and might encourage unhealthy eating behavior among older adults.^14,15^ The prevalence of loneliness has increased among older adults in the US,^16^ which might help explain the decreasing trend in diet quality. In addition, food insecurity, which is not merely the shortage of financial resources to obtain food, is associated with poor diet quality.^17^ The prevalence of food insecurity is estimated to have more than doubled among older US adults during the past 20 years.^18^ Future research is needed to explain the decreasing trend in diet quality among older adults, which would inform the development of public health and nutrition policies and interventions for promoting healthy eating.

The decreasing score in saturated fat intake (ie, higher intake) and the increasing trend in total fat intake in the past 18 years partly reversed the trends observed between 1971 and 2000, when the consumption of fat decreased while the consumption of carbohydrates increased as a proportion of total caloric intake.^19^ These opposing trends could be at least partially due to the shift of dietary guidelines. Since the 1970s, US government guidance has suggested lowering the consumption of fat and saturated fat along with dietary cholesterol from 40% to 30% of total calories owing to the association between these nutrients and increased risk of coronary heart disease.^20^ Overall changes in consumption of carbohydrate-containing foods from 2001 to 2018 were associated with decreasing overall diet quality. In this study, we observed a decrease in mean consumption of whole grains and an increase in consumption of refined grains in both the AHA primary score and the HEI-2015 total score (Table 2). In contrast to the positive change in whole grain consumption identified from 1999 to 2012 among US adults, our study observed an overall decreasing trend in the consumption of whole grains among older adults, particularly between 2015 and 2018. Whole grains are rich in dietary fiber, iron, and many other micronutrients and are recommended as an important component of a healthy diet.^4^ A higher intake of whole grains is associated with a lower risk of many chronic diseases, including cardiovascular disease, cancer, and diabetes.^21^ However, whole grains are more costly than refined grains, creating an additional barrier to consumer access to healthy foods.^22^ Therefore, government and food manufacturers need to provide more affordable healthy foods for older adults, especially those with a limited budget for food.

We found that decreasing dietary quality was more substantial among non-Hispanic White individuals. However, the proportion of older US adults with poor dietary quality increased, and the proportion with ideal diet quality remained low. Older US adults with low levels of income and educational attainment have constantly experienced a poorer diet from 2001 to 2018. Healthier diets cost more than unhealthy diets.^23^ Our findings also explained how the low cost of empty calories relative to the higher cost of nutrient-rich foods exacerbated social inequalities in dietary health. Besides, food budgets in low-income families often fail to ensure a balanced healthy diet. Although the US Department of Agriculture Thrifty Food Plan attempted to recommend food combinations that suit low-income families, the Thrifty Food Plan was later regarded as insufficient.^24^

### Strengths and Limitations

This study has some strengths, including the use of the most recent nationally representative dietary data from the NHANES, providing the most up-to-date dietary profile among older US adults. Thus, our investigation may inform priorities and policies to improve the diets and health of older adults in the US. We provided detailed and comprehensive findings regarding trends in diet quality through examining both the overall diet scores and individual nutrients and food groups. We also investigated the potential differences in dietary intake and quality by key sociodemographic characteristics, allowing for capturing disparities.

This study also has several limitations. First, self-reported dietary 24-hour recall data are subject to measurement error owing to large day-to-day variations in food intake. We used the residual method to reduce measurement error and improve estimates of usual intake. Second, changes in dietary databases and dietary assessment methods during the study period may affect estimated trends in macronutrient intake. We followed the same protocols to derive each macronutrient from different foods across all cycles. Both AHA and HEI analyses using the same dietary database and assessment method showed similar results even though some components were categorized differently. Third, participants with missing data on educational level or income were excluded. The amount of missing data was relatively small and would unlikely affect the generalizability of study findings.

## Conclusions

Despite increases in educational and income levels, overall dietary quality has decreased from 2001 to 2018 based on the AHA and HEI scores among older adults in the US. Older adults have become the fastest-growing segment of the US population; specific attention should be focused on their diets and on diet-related policy to improve their health.

## Notes

Supplement. **eTable 1.** Dietary Components of the American Heart Association (AHA) 2020 Strategic Impact Goals Scoring Standards **eTable 2.** Dietary Components of the Healthy Eating Index (HEI)-2015 and Scoring Standards **eTable 3.** Trends in Estimated Secondary American Heart Association (AHA) Diet Scores by Sociodemographic Characteristics, NHANES Survey Cycles, 2001-2018 **eFigure.** Trends in Mean Diet Quality Score Among US Adults Aged ≥65 Years by NHANES Survey Cycles, 2001-2018
