# Supplementary material for: Trends in Diet Quality Among Older US Adults From 2001 to 2018
Source: JAMA Netw Open. 2022 Mar 11;5(3):e221880. doi: 10.1001/jamanetworkopen.2022.1880 (PMC8917422; doi:10.1001/jamanetworkopen.2022.1880)
Supplement: Supplement. — eTable 1. Dietary Components of the American Heart Association (AHA) 2020 Strategic Impact Goals Scoring Standards eTable 2. Dietary Components of the Healthy Eating Index (HEI)-2015 and Scoring Standards eTable 3. Trends in Estimated Secondary American Heart Association (AHA) Diet Scores by Sociodemographic Characteristics, NHANES Survey Cycles, 2001-2018 eFigure. Trends in Mean Diet Quality Score Among US Adults Aged ≥65 Years by NHANES Survey Cycles, 2001-2018 [file jamanetwopen-e221880-s001.pdf]

Supplementary Online Content

Long T, Zhang K, Chen Y, Wu C. Trends in diet quality among older US adults from 2001 to 2018. *JAMA Netw Open.* 2022;5(3):e221880. doi:10.1001/jamanetworkopen.2022.1880

eTable 1. Dietary Components of the American Heart Association (AHA) 2020 Strategic Impact Goals Scoring Standards
eTable 2. Dietary Components of the Healthy Eating Index (HEI)-2015 and Scoring Standards
eTable 3. Trends in Estimated Secondary American Heart Association (AHA) Diet Scores by Sociodemographic Characteristics, NHANES Survey Cycles, 2001-2018
eFigure. Trends in Mean Diet Quality Score Among US Adults Aged ≥65 Years by NHANES Survey Cycles, 2001-2018

This supplementary material has been provided by the authors to give readers additional information about their work.

© 2022 Long T et al. *JAMA Network Open.*

eTable 1. Dietary Components of the American Heart Association (AHA) 2020 Strategic Impact Goals Scoring Standards

**AHA Components**	**Points Range**	**Scoring Standard**[a]	
		**Max**	**Min**
**Primary Components**[b]	0-50		
Fruits and vegetables[c]	0-10	≥4.5 cups equiv. per day	0
Fish and shellfish	0-10	≥1 oz equiv. per day	0
Whole grains	0-10	≥3 oz equiv. per day	0
Sugar-sweetened beverages	10-0	≤ 5.14 fl oz per day	>16 fl per day
Sodium	10-0	≤ 1500 mg per day	>4500 mg per day
**Secondary Components**[b]	0-80		
Nuts, seeds and legumes[d]	0-10	≥4 servings per day	0
Processed meat	10-0	≤ 0.5 oz equiv. per day	>1.764 oz equiv. per day
Saturated fat	10-0	≤7% energy	>15% energy

[a] Intakes between the minimum and maximum standards are scored proportionately.
[b] All AHA dietary variables were energy-adjusted to 2000kcal/d using the residual method prior to analysis.
[c] According to the AHA 2020 Goals, up to 3 cups/wk (0.42 cups/d) of starchy vegetables (e.g., potatoes, peas, corn) could be included; this maximum was incorporated into the analysis, with higher intake not contributing toward the score. 100% fruit juice could also be included; while its contribution was not capped in the original AHA 2020 Goals and thus not in our score, some organizations recommend no more than 1 serving/d of 100% fruit juice.
[d] A serving of nuts, seeds and legumes is 1-oz equivalent of nuts and seeds or 1⁄2 cup of legume.

© 2022 Long T et al. *JAMA Network Open.*

eTable 2. Dietary Components of the Healthy Eating Index (HEI)-2015 and Scoring Standards

**HEI-2015 Components**	**Points Range**	**Scoring Standard**[a]	
		**Max**	**Min**
**Adequacy Components**			
Total Fruits[b]	0-5	≥0.8 cup equiv. per 1,000 kcal	0
Whole Fruits[c]	0-5	≥0.4 cup equiv. per 1,000 kcal	0
Total Vegetables[d]	0-5	≥1.1 cup equiv. per 1,000 kcal	0
Greens and Beans[d]	0-5	≥0.2 cup equiv. per 1,000 kcal	0
Whole Grains	0-10	≥1.5 oz equiv. per 1,000 kcal	0
Dairy[e]	0-10	≥1.3 cup equiv. per 1,000 kcal	0
Total Protein Foods[f]	0-5	≥2.5 oz equiv. per 1,000 kcal	0
Seafood and Plant Proteins[e,g]	0-5	≥0.8 oz equiv. per 1,000 kcal	0
Fatty Acids	0-10	(PUFAs + MUFAs)/SFAs ≥2.5	(PUFAs + MUFAs)/SFAs ≤1.2
**HEI-2015 Moderation**			
Refined Grains	10-0	≤1.8 oz equiv. per 1,000 kcal	≥4.3 oz equiv. per 1,000 kcal
Sodium	10-0	≤1.1 grams per 1,000 kcal	≥2.0 grams per 1,000 kcal
Added Sugars	10-0	≤6.5% of energy	≥26% of energy
Saturated Fats	10-0	≤8% of energy	≥16% of energy

[a] Intakes between the minimum and maximum standards are scored proportionately.
[b] Includes 100% fruit juice.
[c] Includes all forms except juice.
[d] Includes legumes (beans and peas)
[e] Includes all milk products, such as fluid milk, yogurt, and cheese, and fortified soy beverages.
[f] Includes legumes (beans and peas)
[g] Includes seafood, nuts, seeds, soy products (other than beverages), and legumes (beans and peas).
[h] Ratios of poly-and monosaturated fatty acids (PUFAs and MUFAs) to saturated fatty acids (SFAs).

© 2022 Long T et al. *JAMA Network Open.*

eTable 3. Trends in Estimated Secondary American Heart Association (AHA) Diet Scores by Sociodemographic Characteristics, NHANES Survey Cycles, 2001-2018

Survey-weighted mean, 95%CI												
		**2001-2002**	**2003-2004**	**2005-2006**	**2007-2008**	**2009-2010**	**2011-2012**	**2013-2014**	**2015-2016**	**2017-2018**	**P for trend**	**P for interaction**
**AHA Secondary score**												
**Age group, y**												
		33.97	31.26	36.46	31.54	34.92	32.74	33.68	33.44	29.51	0.02	Ref
	65-69	(32.41-35.53)	(29.56-32.96)	(34.71-38.21)	(29.99-33.09)	(33.40-36.45)	(30.55-34.93)	(32.23-35.13)	(31.51-35.37)	(27.45-31.57)		
		34.01	33.46	38.05	33.45	35.52	34.06	35.24	33.87	31.45	0.008	0.93
	70-74	(32.53-35.49)	(32.02-34.91)	(36.37-39.73)	(32.04-34.86)	(34.30-36.74)	(32.57-35.56)	(33.52-36.96)	(32.07-35.67)	(29.84-33.07)		
		34.06	34.19	37	34.12	35.38	32.14	34.09	33.62	31.82	0.006	0.85
	75-79	(32.31-35.82)	(32.59-35.79)	(35.10-38.89)	(32.78-35.46)	(33.91-36.84)	(30.56-33.71)	(32.21-35.98)	(31.65-35.58)	(29.96-33.67)		
		35.38	35.57	40.47	35.19	38.34	34.45	37.14	35.24	30.29	<0.001	0.14
	≥ 80	(34.16-36.61)	(34.46-3.67)	(39.27-41.68)	(33.97-36.41)	(37.23-39.45)	(33.21-35.69)	(35.73-38.56)	(34.06-36.43)	(28.89-31.69)		
**Sex**												
		35.58	34.24	39.89	34.39	37.46	34.91	35.91	35.61	31.78	<0.001	0.88
	Female	(34.57-36.59)	(33.19-35.29)	(38.78-41.00)	(33.43-35.34)	(36.58-38.35)	(33.74-36.08)	(34.88-36.94)	(34.34-36.88)	(30.53-33.03)		
		32.69	32.39	35.21	32.19	34.17	31.62	33.69	31.79	29.19	<0.001	Ref
	Male	(31.56-33.82)	(31.28-33.50)	(33.96-36.47)	(31.12-33.27)	(33.07-35.26)	(30.25-32.99)	(32.39-34.99)	(30.42-33.16)	(27.72-30.66)		
**Race/Ethnicity**												
		36.27	36.39	38..83	36.78	37.87	33.64	35.66	37.27	33.99	0.04	Ref
	Non-Hispanic White	(33.75-38.80)	(34.43-38.35)	(36.32-41.35)	(35.30-38.26)	(36.53-39.21)	(31.64-35.63)	(33.91-37.41)	(36.04-38.50)	(32.11-35.87)		
		42.5	42.14	45.53	41.78	40.95	36.7	41.26	36.22	36.9	<0.001	0.006
	Non-Hispanic Black	(33.20-51.80)	(37.94-46.34)	(40.65-50.41)	(7.02-46.53)	(37.86-44.04)	(32.69-40.71)	(38.78-43.74)	(33.30-39.14)	(34.75-39.05)		
		34.06	32.92	37.62	32.92	35.64	33.19	34.47	33.47	29.86	<0.001	0.26
	Hispanics	(33.27-34.86)	(32.12-33.72)	(36.73-38.51)	(32.16-33.68)	(34.88-36.40)	(32.20-34.18)	(33.56-35.37)	(32.40-34.55)	(28.79-30.94)		
**Marital Status**												
	Married/Living with partner	33.98	33.83	37.56	33.35	35.7	33.32	34.89	33.73	30.33	<0.001	Ref
		(32.95-35.02)	(32.80-34.86)	(36.40-38.71)	(32.34-34.36)	(34.77-36.64)	(32.09-34.55)	(33.80-35.97)	(32.43-35.03)	(29.07-31.59)		
	Widowed	35.03	33.47	38.94	34.51	36.9	33.99	35.62	34.11	31.58	0.003	0.60

© 2022 Long T et al. *JAMA Network Open.*

		(33.73-36.33)	(32.22-34.72)	(37.44-40.44)	(33.29-35.72)	(35.76-38.05)	(32.52-35.46)	(34.02-37.22)	(32.25-35.97)	(29.71-33.45)		
	Divorced/Separated	33.96	30.76	36.77	32.38	35.01	32.23	32.61	34.26	30.83	0.17	0.33
		(31.36-36.55)	(28.23-33.28)	(34.23-39.30)	(30.56-34.20)	(32.54-37.49)	(29.65-34.81)	(30.68-34.53)	(32.43-36.08)	(28.36-33.31)		
	Never married	33.92	34.35	41.6	32.07	37.77	36.04	40.09	36.69	27.51	0.47	0.67
		(29.02-38.82)	(29.03-39.66)	(38.07-45.14)	(28.00-36.13)	(34.93-40.61)	(32.92-39.16)	(36.03-44.16)	(31.36-42.02)	(23.78-31.23)		
**Education Level**												
	< high school diploma	33.28	31.22	37.98	31.94	35.28	32.44	34.32	35.75	30.09	0.74	Ref
		(31.94-34.63)	(30.00-32.43)	(36.57-39.39)	(30.75-33.12)	(34.15-36.40)	(31.15-33.74)	(32.63-36.02)	(34.32-37.18)	(27.76-32.42)		
	High school graduate or GED	33.68	33.88	37.05	33.41	34.69	32.15	34.35	33.18	28.62	<0.001	0.001
		(32.21-35.15)	(32.47-35.29)	(35.42-38.67)	(32.13-34.68)	(33.33-36.05)	(30.16-34.14)	(32.78-35.92)	(31.24-35.13)	(26.89-30.36)		
	Some college	35.02	33.08	38.06	32.84	36.65	33.36	34.24	32.67	31.66	<0.001	0.02
		(33.56-36.49)	(31.37-34.79)	(36.33-39.80)	(31.18-34.51)	(35.19-38.11)	(31.62-35.10)	(32.71-35.77)	(31.10-34.24)	(29.95-33.37)		
	≥ College degree	35.87	36.71	38.84	36.11	37.27	35.48	36.29	35.01	31.52	<0.001	0.001
		(34.00-37.74)	(34.78-38.65)	(36.69-40.99)	(34.40-37.83)	(35.66-38.88)	(33.40-37.56)	(34.65-37.92)	(32.86-37.16)	(29.67-33.36)		
**Ratio of family income to poverty level**												
	<1.30	33.98	31.87	38.22	33.12	35.84	33.05	34.88	34.16	31.4	0.27	Ref
		(32.50-35.46)	(30.36-33.39)	(36.35-40.09)	(31.79-34.45)	(34.55-37.12)	(31.63-34.47)	(33.19-36.57)	(32.54-35.79)	(29.57-33.23)		
	1.30 to <1.85	32.05	31.1	36.2	33.15	35.53	30.73	34.75	32.26	31.43	0.44	0.88
		(29.98-34.11)	(29.16-33.04)	(34.22-38.17)	(31.45-34.85)	(33.74-37.33)	(27.60-33.85)	(32.47-37.04)	(30.49-34.02)	(29.82-33.03)		
	1.85 to <3.00	33.92	33.82	38.45	32.62	34.79	33.78	34.5	32.78	28.79	<0.001	0.009
		(32.49-35.36)	(32.24-35.39)	(36.75-40.15)	(31.21-34.03)	(33.40-36.18)	(31.81-35.74)	(32.85-36.16)	(30.96-34.59)	(26.73-30.85)		
	≥ 3.00	35.23	34.82	37.74	34.26	36.4	33.91	35.12	33.98	30.69	<0.001	0.009
		(33.82-36.65)	(33.45-36.19)	(36.20-39.27)	(32.86-35.67)	(35.16-37.64)	(32.39-35.43)	(33.79-36.46)	(32.18-35.78)	(29.06-32.31)		

Abbreviations: GED, general equivalency diploma; NHANES, National Health and Nutrition Examination Survey.

[a] Data were weighted to be nationally representative. P for trend was estimated by treating survey-cycle as a continuous variable in a survey-weighted logistic regression model. P for interaction was calculated using the regression model for an interaction term between survey-cycle and sociodemographic subgroups.

© 2022 Long T et al. *JAMA Network Open.*

eFigure. Trends in Mean Diet Quality Score Among US Adults Aged ≥65 years by NHANES Survey Cycles, 2001-2018

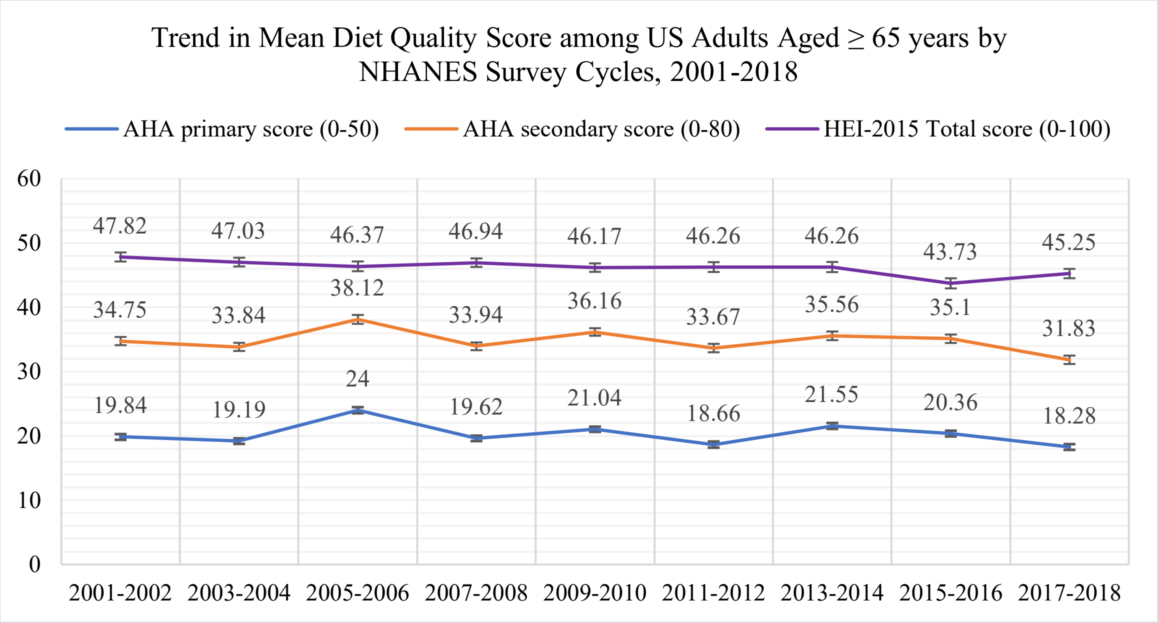

© 2022 Long T et al. *JAMA Network Open.*
